# Roles of AP-2 in Clathrin-Mediated Endocytosis

**DOI:** 10.1371/journal.pone.0010597

**Published:** 2010-05-12

**Authors:** Emmanuel Boucrot, Saveez Saffarian, Rongying Zhang, Tomas Kirchhausen

**Affiliations:** Department of Cell Biology, Harvard Medical School and Immune Disease Institute at Children's Hospital, Boston, Massachusetts, United States of America; University of California, United States of America

## Abstract

**Background:**

The notion that AP-2 clathrin adaptor is an essential component of an endocytic clathrin coat appears to conflict with recent observations that substantial AP-2 depletion, using RNA interference with synthesis of AP-2 subunits, fails to block uptake of certain ligands known to internalize through a clathrin-based pathway.

**Methodology/Principal Findings:**

We report here the use of *in vivo* imaging data obtained by spinning-disk confocal microscopy to study the formation of clathrin-coated structures at the plasma membranes of BSC1 and HeLa cells depleted by RNAi of the clathrin adaptor, AP-2. Very few clathrin coats continue to assemble after AP-2 knockdown. Moreover, there is a total absence of clathrin-containing structures completely lacking AP-2 while all the remaining coats still contain a small amount of AP-2. These observations suggest that AP-2 is essential for endocytic coated-pit and coated-vesicle formation. We also find that AP-2 knockdown strongly inhibits light-density lipoprotein (LDL) receptor-mediated endocytosis, as long as cells are maintained in complete serum and at 37°C. If cells are first incubated with LDL at 4°C, followed by warming, there is little or no decrease in LDL uptake with respect to control cells. LDL uptake at 37°C is also not affected in AP-2 depleted cells first deprived of LDL by incubation with either serum-starved or LDL-starved cells for 24 hr. The LDL-deprived cells display a significant increase in endocytic structures enriched on deeply invaginated tubes that contain LDL and we suggest that under this condition of stress, LDL might enter through this alternative pathway.

**Conclusions/Significance:**

These results suggest that AP-2 is essential for endocytic clathrin coated-pit and coated-vesicle formation. They also indicate that under normal conditions, functional endocytic clathrin coated pits are required for LDL internalization. We also show that under certain conditions of stress, cells can upregulate alternative endocytic structures with the potential to provide compensatory trafficking pathways.

## Introduction

Heterotetrameric clathrin adaptor complexes (APs) are major components of clathrin-coated vesicles, second in abundance only to clathrin itself [1]. They link the clathrin lattice with the underlying membrane, primarily through specific interactions with the headgroups of PI(4,5)P_2_ or PI4P and through recognition of sorting signals on cytosolic segments of membrane proteins destined for inclusion as cargo (reviewed in [2]). In higher eukarotic cells, endocytic clathrin coats originating at the plasma membrane contain AP-2 adaptors comprising α-, β2-, μ2-, and σ2-adaptin subunits. The absence of detectable endocytic clathrin structures devoid of AP-2 in cells with normal levels of AP-2 and the correlated dynamics of clathrin and AP-2 recruitment into coated pits both suggest that AP-2 is essential for endocytic clathrin coat formation [3], [4].

The notion that AP-2 is an essential component of an endocytic clathrin coat appears to conflict with the recent observation that substantial AP-2 depletion, using RNA interference with synthesis of μ2-, β2- or α-adaptin, fails to block uptake of certain ligands known to internalize through a clathrin-based pathway: light-density lipoprotein (LDL) [5]–[8], epidermal growth factor (EGF) [5], [8], [9] and influenza virus [8]. The issue remains unresolved, however, for several reasons. First, AP-2 was not totally eliminated in the RNAi experiments ([5], [6], [9]; no data shown in [7], [8]). Second, although AP-2 depletion results in a substantial decrease in the number of clathrin coats detected by electron microscopy at the cell surface of fixed cells [5], [9], it is possible that the remaining coats contain residual AP-2 left by the inevitably incomplete knockdown. Third, measurement of LDL or LDL-receptor (LDLR) uptake was carried out with cells not maintained in normal culture conditions (full serum and 37°C) but instead pre-incubated with lipoprotein-depleted serum or incubated at 4°C during ligand binding, followed by warming to 37°C [5]–[8]. As EGF uptake in AP-2 depleted cells is reduced if cells remain at 37°C throughout the experiment, but not if the binding step is performed at 4°C [10], the detailed protocol may matter. Fourth, in the case of influenza uptake [8], the reported assay followed a late step in viral infection, rather than measuring endocytosis directly, and the work used BSC1 cells, which like other non-polarized cells can sustain equally good levels of infection through clathrin-dependent and clathrin-independent entry pathways [11].

We address here two principal questions. First, after AP-2 depletion, can we find clathrin coats devoid of AP-2, or does the reduced number of coats that form always contain a detectable amount of the residual AP-2 not eliminated by the RNAi treatment? Second, what happens to receptor-mediated endocytosis of LDL in AP-2 depleted cells, if the cells remain in physiological conditions (complete serum and 37°C) throughout the experiment? Using live-cell fluorescence microscopy imaging, we confirm the severe reduction in the number of clathrin coats forming in cells severely depleted of AP-2. We find, however, that all of the remaining coats always contain a small amount of AP-2. The reduction in the number of endocytic clathrin coats parallels the reduction in receptor mediated LDL uptake in AP-2 depleted cells kept in physiological conditions. In contrast, the apparently normal uptake of LDL observed in AP-2 depleted cells exposed to LDL-depleted serum appears to be due to the upregulation of an extensive network of tubular structures emanating from the plasma membrane.

## Methods

### Cells, plasmids, transfections and reagents

BSC1 (BS-C-1; ATCC CCL-26) and HeLa (ATCC CCL-2) cells were grown at 37°C with 5% CO_2_ and 100% humidity as adherent cells in DMEM supplemented with 10% fetal bovine serum (FBS). The generation and characterization of BSC1 and HeLa cell lines stably expressing EGFP-fusion chimeras of LCa or σ2 were described previously [3], [12]. We have generated cell lines stably expressing CD8-LDLR (a gift from M. Robinson) by transfection with the plasmid (see below) followed by selection and maintenance with complete medium supplemented with geneticin (G418, 0.5–0.7 mg/ml). Transient transfections of Tomato-LCa (previously described in [12]) on σ2-EGFP stable cell lines were carried out using FuGENE 6 (Roche Diagnostics, Indianapolis, IN) for HeLa cells and Lipofectamine 2000 (Invitrogen, Carlsbad, CA) for BSC1 cells and the cells were studied 24–48 h after.

The following reagents were used: human transferrin labeled with Alexa Fluor 594 (Tf-A594) or Alexa Fluor 647 (Tf-A647), FM 1-43 (Molecular Probes, Eugene, OR); LDL-Cy5 was prepared by labeling LDL (a gift from Prof. Angelo Scanu, University of Chicago) with a Cy5-labelling kit (Molecular Probes, Eugene, OR or Amersham GE Healthcare, UK). The following antibodies were used: mouse monoclonal anti-human CD8 clone UCHT-4 (Sigma, Saint Louis, MO), mouse anti-actin (Sigma, Saint Louis MO), mouse anti μ2-adaptin (Transduction Laboratories) and mouse anti α-adaptin (BD Biosciences, San Jose CA).

### Small interfering RNA

**Figure 1:**
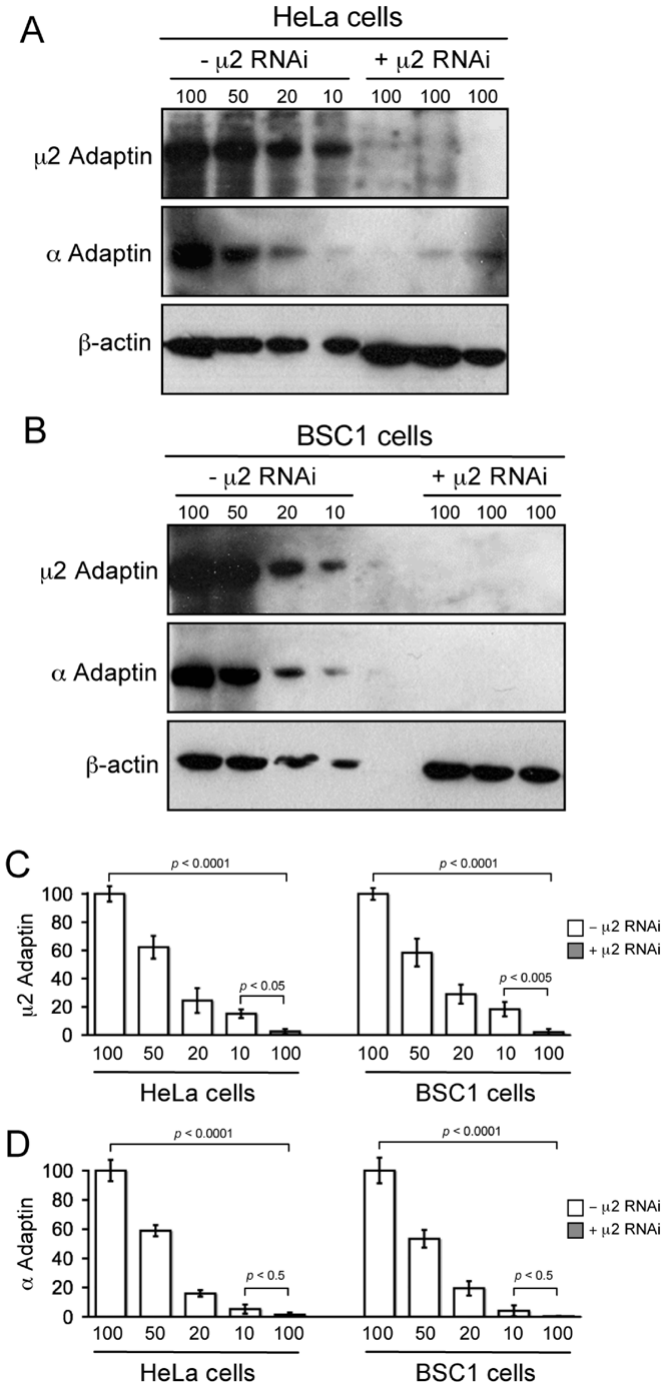
RNAi Depletion of AP-2. (**A**, **B**) Immunoblots of cell extracts from HeLa or BSC1 cells treated with for 72 h in presence or absence of μ2- siRNA oligos and probed with antibodies specific for μ2-adaptin, α-adaptin or β-actin (used as loading control). Serial dilutions (30 µl (100%), 15 µl (50%), 6 µl (20%) and 3 µl (10%)) of the control cell extracts were processed for western blots; 30 µl (100%) were used for 3 independent μ2-RNAi cell extracts. (**C**,**D**) Residual μ2-adaptin and α-adaptin in each cell type, obtained from three independent RNAi experiments. Each band was background-corrected, normalized to the corresponding intensity of the actin band, then expressed as percent of its value with respect to the 100% loading.

**Figure 2:**
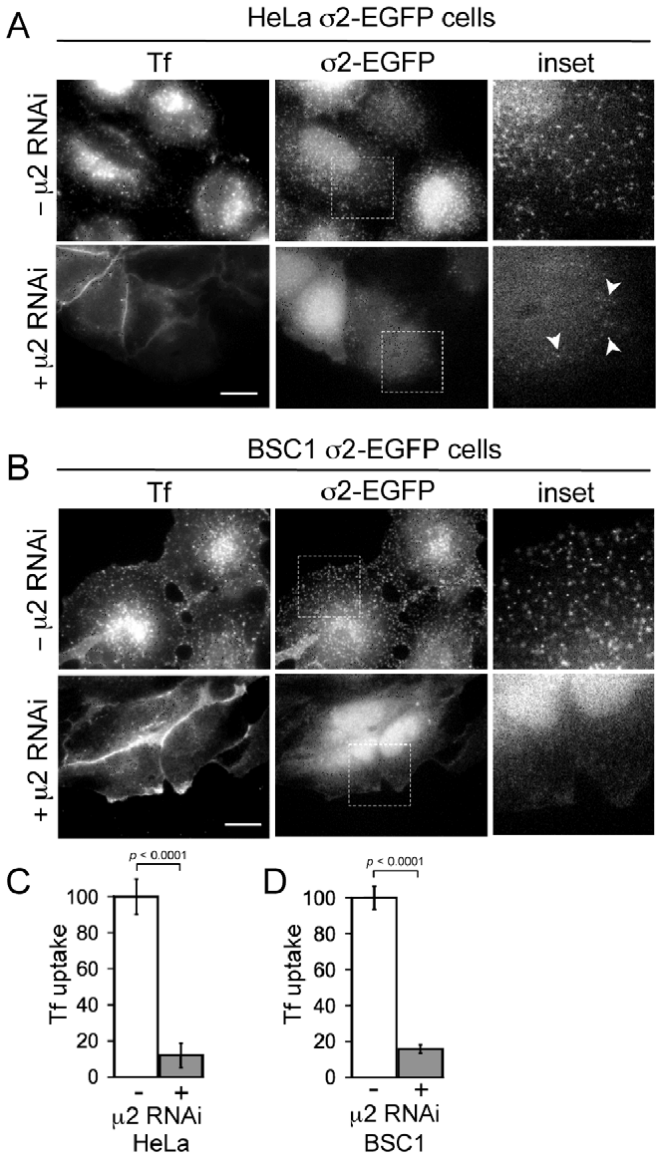
Effect of AP-2 depletion on the surface distribution of AP-2 and on the uptake of transferrin. (**A**, **B**) Control (-μ2 RNAi) and treated (+ μ2 RNAi) HeLa and BSC1 cells stably expressing σ2-EGFP were incubated at 37°C with 10 µg/ml of Tf-A594 for 10 min, followed by a quick rinse at 4°C to remove unbound transferrin, fixed with 3.7% PFA, and imaged in 3D with wide-field illumination. Representative views of the plane closest to the coverslip (σ2-EGFP) and of an equatorial plane (Tf) show a decrease in punctate σ2-EGFP signals at the cell membrane and inhibition of transferrin uptake. Insets correspond to representative boxed regions. Arrowheads mark the few remaining AP-2 spots in Tf-negative cells. Bar, 20 µm. (**C**, **D**) Histrograms showing the amount, relative to controls, of internalized transferrin obtained from the total fluorescence signal of Tf-A594 in the 3D stacks. The remaining signals were 11.9±6.9% (*p*<0.0001, 10 control and 14 AP-2 depleted HeLa cells) and 15.8±2.5% (*p*<0.0001, 15 control and 22 AP-2 depleted BSC1 cells), respectively.

BSC1 or HeLa cells in 6-well plates were transfected using Oligofectamine (Invitrogen Carlsbad, CA) with 6 nM siRNA specific for μ2-adaptin [5] purchased from Dharmacon, Inc. (Lafayette, CO), followed by a second transfection 24 h later with 6 nM siRNA; experiments were conducted after an additional 48 interval (for Figure 1). Similar results were obtained when BSC1 or HeLa cells in 6-well plates were subjected to two successive transfections, performed 48 h apart, with 10 nM siRNA each time. Cells were plated on glass coverslips 36 h after the second transfection and studied 12 h after (for Figure 2–7). Control cells were treated equally but with no μ2 siRNA.

**Figure 3 pone-0010597-g003:**
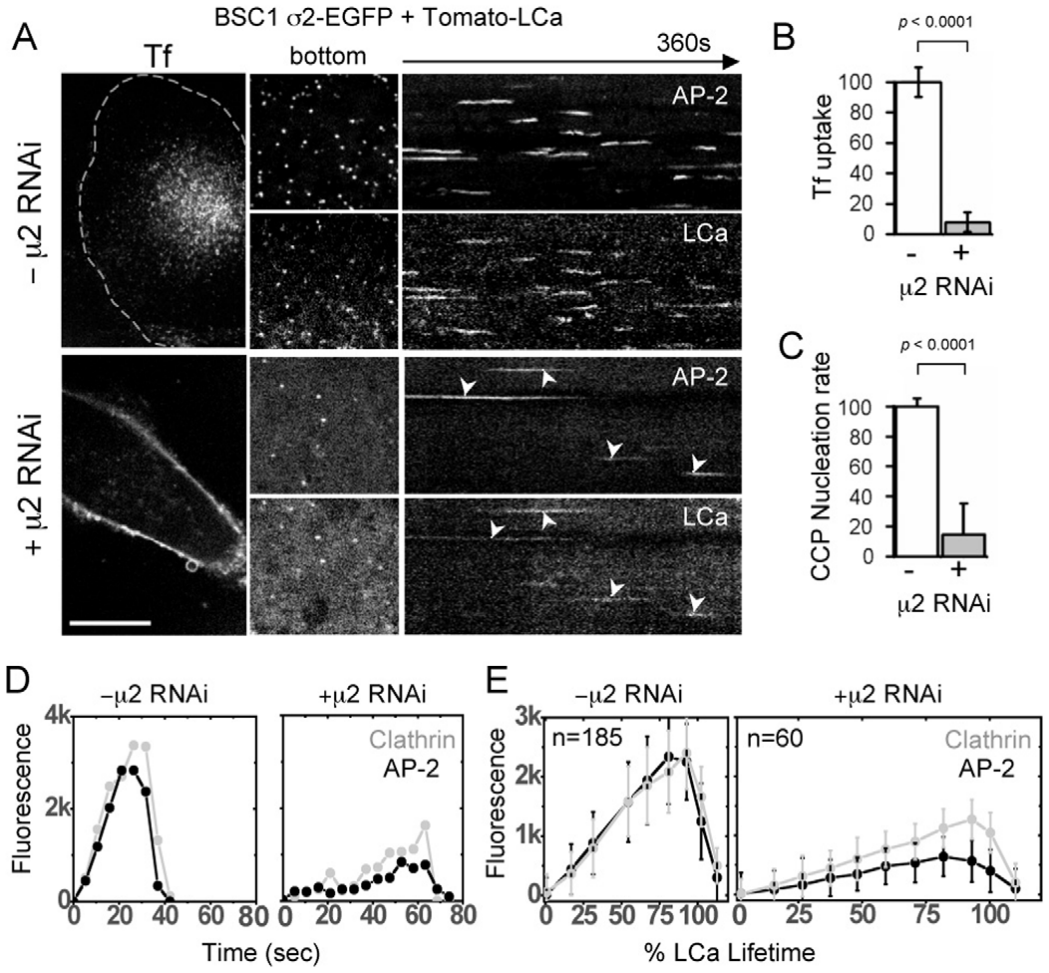
Effect of AP-2 depletion on the formation of clathrin-coated structures at the plasma membrane of BSC1 cells. (**A**) Time-series acquired with a spinning disk confocal microscope. The fluorescent assemblies were tagged with transiently expressed clathrin Tomato-LCa and with stably expressed AP-2 σ2-EGFP in the presence or absence of μ2-siRNA. The left hand panels at the left correspond to central optical sections of cells incubated with 10 µg/ml Tf-A647 for 10 min at 37°C. They show accumulation of Tf in endosomal structures in the control cells and Tf on the plasma membrane in AP-2 depleted cells (Bar, 10 µm). The central panels are snapshots and the right panels are kymographs from representative time series recorded every 2 s (500 ms exposures) for 360 s from the adherent (bottom) surface of the cells. The majority of the structures are dynamic and relatively short lived in control cells; their number and their clathrin and AP-2 content are greatly decreased in AP-2 depleted cells. (**B**) Transferrin (Tf) uptake in AP-2 depleted cells. The amount (in fluorescence arbitrary units) of internalized transferrin in each cell was obtained at the end of the time series from the total fluorescence signal of Tf-A647 in a 3D stack (see Methods). Tf uptake decreased to 7.8±6.4% of its control value (*p*<0.0001, from 3 control and 3 AP-2 depleted cells). (**C**) Nucleation rate (normalized to control levels) of clathrin-coated pits and vesicles (CCP) tagged with Tomato-LCa forming at the adherent (bottom) surface of BSC1 cells in the absence and presence of μ2-siRNA. The actual nucleation rate drop from 3.9±0.2 to 0.6±0.1 coated pits per 10^4^ µm^2^.s^−1^ in cells depleted of AP-2 (*p*<0.0001; 4 controls and 4 AP-2 depleted cells). (**D**) Fluorescence intensity profiles of an individual clathrin coat tagged with clathrin Tomato-LCa and AP-2 σ2-EGFP forming in the presence (+ μ2-RNAi) and absence (- μ2-RNAi) of μ2-siRNA. (**E**) Average fluorescence intensity profiles of 60 clathrin coats tagged with clathrin Tomato-LCa and AP-2 σ2-EGFP forming in 3 cells depleted of AP-2 (+ μ2 RNAi) relative to 185 coats in 3 control cells (- μ2 RNAi). These cells expressed comparable amounts of clathrin tagged with Tomato-LCa as judged by the similarity in clathrin fluorescence determined associated with their perinuclear regions. All structures forming in AP-2 depleted cells contain statistically lower amounts of clathrin and AP-2 (*p*<0.0001; see details in Figure 4).

**Figure 4 pone-0010597-g004:**
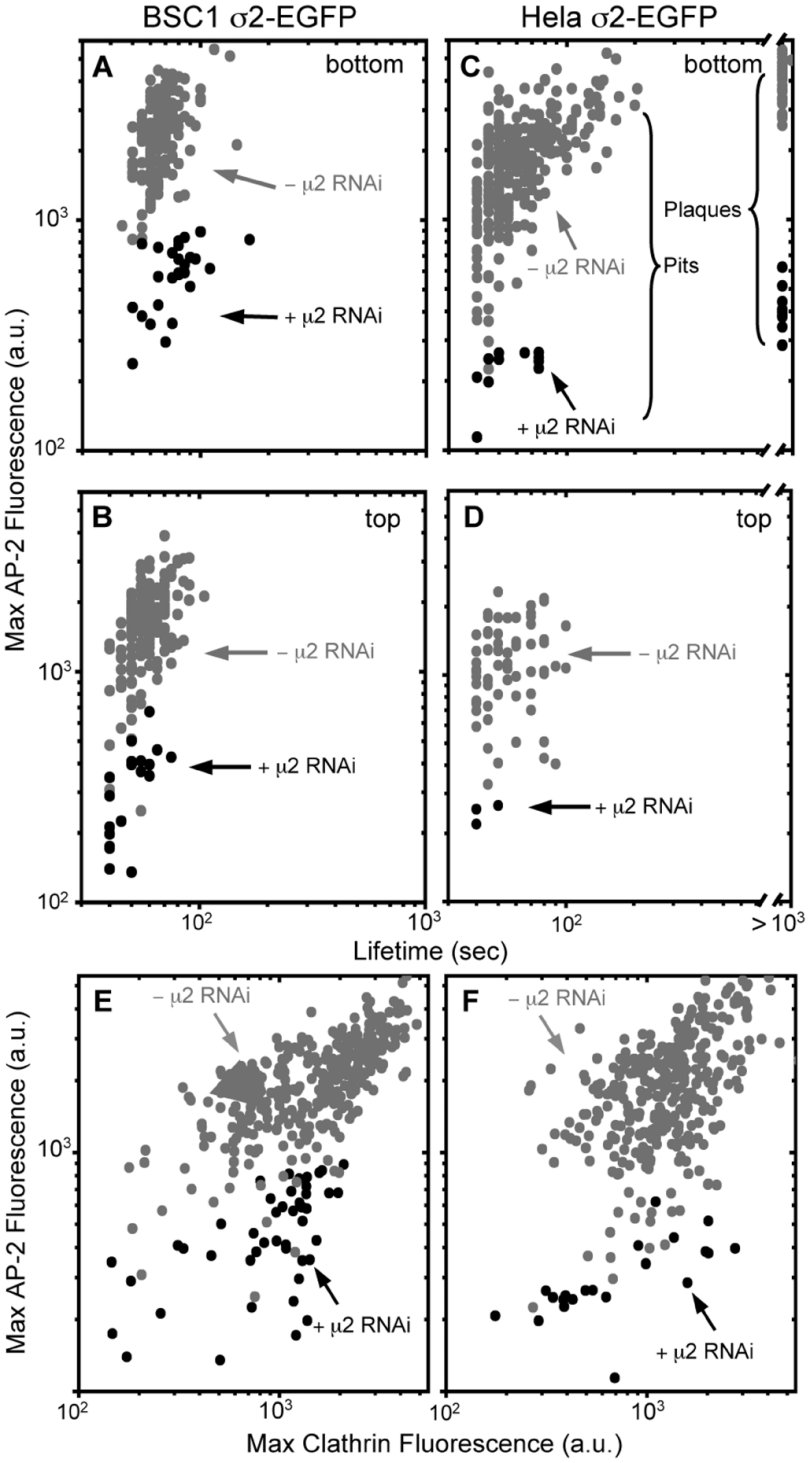
Effect of AP-2 depletion on sizes and lifetimes of clathrin-coats forming at the cell surface. The double logarithmic plots show the maximum fluorescence intensity (prior to dissolution) and the lifetimes of every fluorescent spot containing clathrin and AP-2 recorded at the adhered (bottom) or free (top) plasma membrane of BSC1 and HeLa cells stably expressing AP-2 σ2-EGFP together with transient expression of Clathrin Tomato-LCa. The spots are color-coded to distinguish the effect of AP-2 depletion by the presence for 96 hr of siRNA specific for μ2-adaptin (black; +μ2 RNAi) from those in the absence of siRNA (gray; -μ2 RNAi) on the properties of endocytic clathrin coats. The data derive from time series of 1000 s duration recorded every 5 s (100 ms exposures) at 37°C acquired from three different cells for each category. The data was acquired using a spinning disk confocal microscope. (**A**–**D**) Effect of AP-2 depletion on the content of AP-2 and the lifetimes of endocytic clathrin coated structures. (**A**, **B**) Maximum fluorescence intensity of AP-2 in clathrin-coated structures are 2491±842 (n = 186) and 1692±645 (n = 150) in the bottom and top of three control BSC1 cells and 600±182 (n = 25) and 330±141 (n = 18) in the corresponding surfaces of three AP-2 depleted cells. Their lifetimes are 67±12 and 60±11 s for control cells, and 79±20 and 51±11 s for AP-2 depleted cells, respectively. (**C**, **D**) Maximum fluorescence intensity of AP-2 in clathrin-coated structures are 1906±873 (n = 296) and 1118±472 (n = 67) in the bottom and top of three control HeLa cells and 231±42 (n = 11) and 247±24 (n = 3) in the corresponding surfaces of AP-2 depleted cells. Their life times are 66±28 and 44±6 s for control cells, and 59±15 and 44±6 s for three AP-2 depleted cells. In each case, the difference in maximum fluorescence intensity is statistically significant smaller in AP-2 depleted cells (*p*<0.0001). (**E**) Effect of AP-2 depletion in the content of clathrin Tomato-LCa and AP-2 σ2-EGFP in each of the endocytic clathrin coated structures of BSC1 cells shown in panels A and B. The maximum fluorescence intensities are 1789±1008 (n = 336) and 1020±489 (n = 43) for clathrin and 2134±857 and 486±212 for AP-2 in control and AP-2 depleted BSC1 cells, respectively. In all cases, the decrease in the number of coated structures and the fluorescence intensity due to AP-2 depletion is statistically significant (*p*<0.0001). (**F**) Effect of AP-2 depletion in the content of clathrin Tomato-LCa and AP-2 σ2-EGFP in each of the endocytic clathrin coated structures of HeLa cells shown in panels C and D. The maximum fluorescence intensities are 1326±686 (n = 363) and 422±146 (n = 14) for clathrin and 1906±874 and 231±43 for AP-2 in control and AP-2 depleted HeLa cells, respectively. In all cases, the decrease in the number of coated structures and the fluorescence intensity due to AP-2 depletion is statistically significant (*p*<0.0001).

**Figure 5 pone-0010597-g005:**
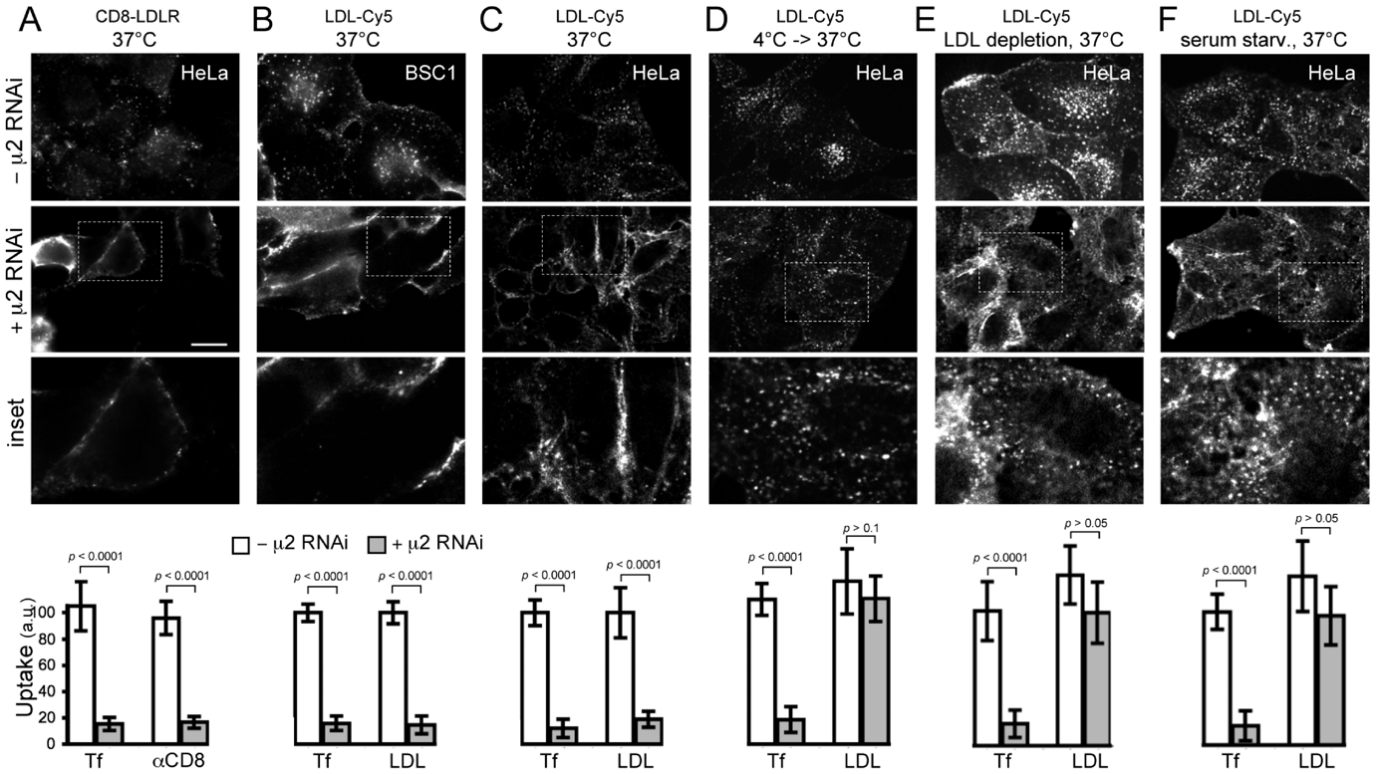
Effect of LDL or serum starvation and of low temperature on the inhibition of LDL uptake in cells depleted of AP-2. Fluorescent 3D image stacks spanning the complete cell were obtained with a spinning disk confocal microscope; the middle plane is shown as a reference. Bar, 20 µm. Insets correspond to representative boxed regions from AP-2 depleted cells. In each experiment shown, control (- μ2-RNAi) and AP-2 depleted (+ μ2-RNAi) cells were incubated for 10 min at 37°C with ligands specific for the experiment as listed below, quickly washed at 4°C to remove unbound ligands, and fixed (see Methods). Histograms show uptake of Tf and LDL (or CD8-LRLR in panel A) from 20 control cells (white bars) and 20 AP-2 depleted cells (gray bars), in 3 independent experiments. (**A**) HeLa cells stably expressing CD8-LDLR. The 10 min incubation included 10 µg/ml Tf-A594 and a monoclonal antibody specific for the ectodomain of human CD8. The reduction in uptake is from 100 to 15.4±5.0 and to 16.7±4.5 a.u. for Tf and CD8-LDL, respectively (*p*<0.0001 in both cases). (**B**) BSC1 cells. The 10 min incubation included 10 µg/ml Tf-A594 and 0.72 µg/ml LDL-Cy5. The reduction in uptake is from 100 to 15.8±5.6 and to 14.6±6.8 a.u. for Tf and LDL, respectively (*p*<0.0001 in both cases). (**C**) HeLa cells. The 10 min incubation included 10 µg/ml Tf-A594 and 0.72 µg/ml LDL-Cy5. The reduction in uptake is from 100 to 11.9±6.9 and 18.9±6.1 a.u. for Tf and LDL, respectively (p<0.0001 in both cases). (**D**) HeLa cells, preincubated for 1 h at 4°C with 10 µg/ml Tf-A594 and 0.72 µg/ml LDL-Cy5, then transferred to 37°C for 10 min. The reduction in Tf uptake is from 100 to 18.7±9.9 a.u. (*p*<0.0001); there is no reduction in LDL uptake (110.7±17.4, *p*>0.05). (**E**) HeLa cells, preincubated for 24 h in medium supplemented with LDL-deficient serum, then for 10 min (at 37°C) with 10 µg/ml Tf-A594 and 0.72 µg/ml LDL-Cy5. The reduction in Tf uptake is from 100 to 17.2±10.7 a.u. (*p*<0.0001); there is no reduction in LDL uptake (103±23.9, *p*>0.05). (**F**) HeLa cells, preincubated for 6 h in serum-depleted medium (0.1% serum), then for 10 min (at 37°C) in normal medium with 10 µg/ml Tf-A594 and 0.72 µg/ml LDL-Cy5. The reduction in Tf uptake is from 100 to 14.6±11.3 a.u. (*p*<0.0001); there is no reduction in LDL uptake (98.0±22.0, *p*>0.05).

**Figure 6 pone-0010597-g006:**
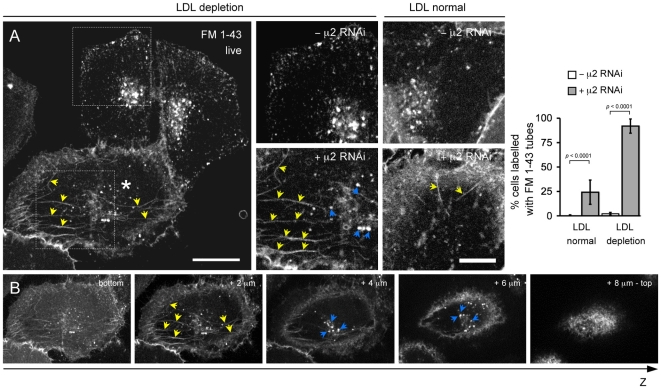
Enrichment of endocytic tubules labeled with FM 1-43 dye in HeLa cells depleted of AP-2 and LDL. (**A**) Effect of AP-2 depletion on the enrichment of tubular structures labeled with FM 1-43 fluorescent dye. The experiment was carried out on a mixture of HeLa cells depleted (+ μ2 RNAi) or not (-μ2 RNAi) of AP-2 by treatment with RNAi specific for μ2. Cells were imaged 24 hrs after plating in medium supplemented with 10% FCS (LDL normal) or with LDL-depleted serum (LDL depletion) using a spinning-disk confocal microscope. Cells depleted of AP-2 (marked with an asterisk ‘*’) were identified by the lack of internalized fluorescent Tf following a brief incubation for 5 min with 10 µg/ml Tf-A594 (*not shown*). Cells were then incubated with 5 µg/ml FM 1-43 for 5 min and imaged live. Yellow arrows in the inset highlight the appearance in the AP-2 depleted cell of tubular structures containing FM 1-43. Blue arrows in the insets highlight spots corresponding to endosomes. Images are representative of 100 random cells analyzed acquired for each category from 3 independent experiments. Bar, 20 µm (10 µm in the insets). The histograms show the fraction of cells containing tubes labeled with FM 1-43 observed in 100 Tf-negative cells selected randomly from the imaging fields. Data for each category were obtained from 3 experiments performed independently. Similar results were obtained using BSC1 cells (*not shown*). (**B**) Images from a Z-stack of consecutive optical planes acquired every 0.3 µm. They highlight the presence of intracellular tubular structures labeled with the FM 1-43-dye (yellow arrows) added to the medium. The tubular structures are contained within the cellular boundaries and are therefore located inside the cell. The images also indicate the intracellular location of endosomal spots (blue arrows).

**Figure 7:**
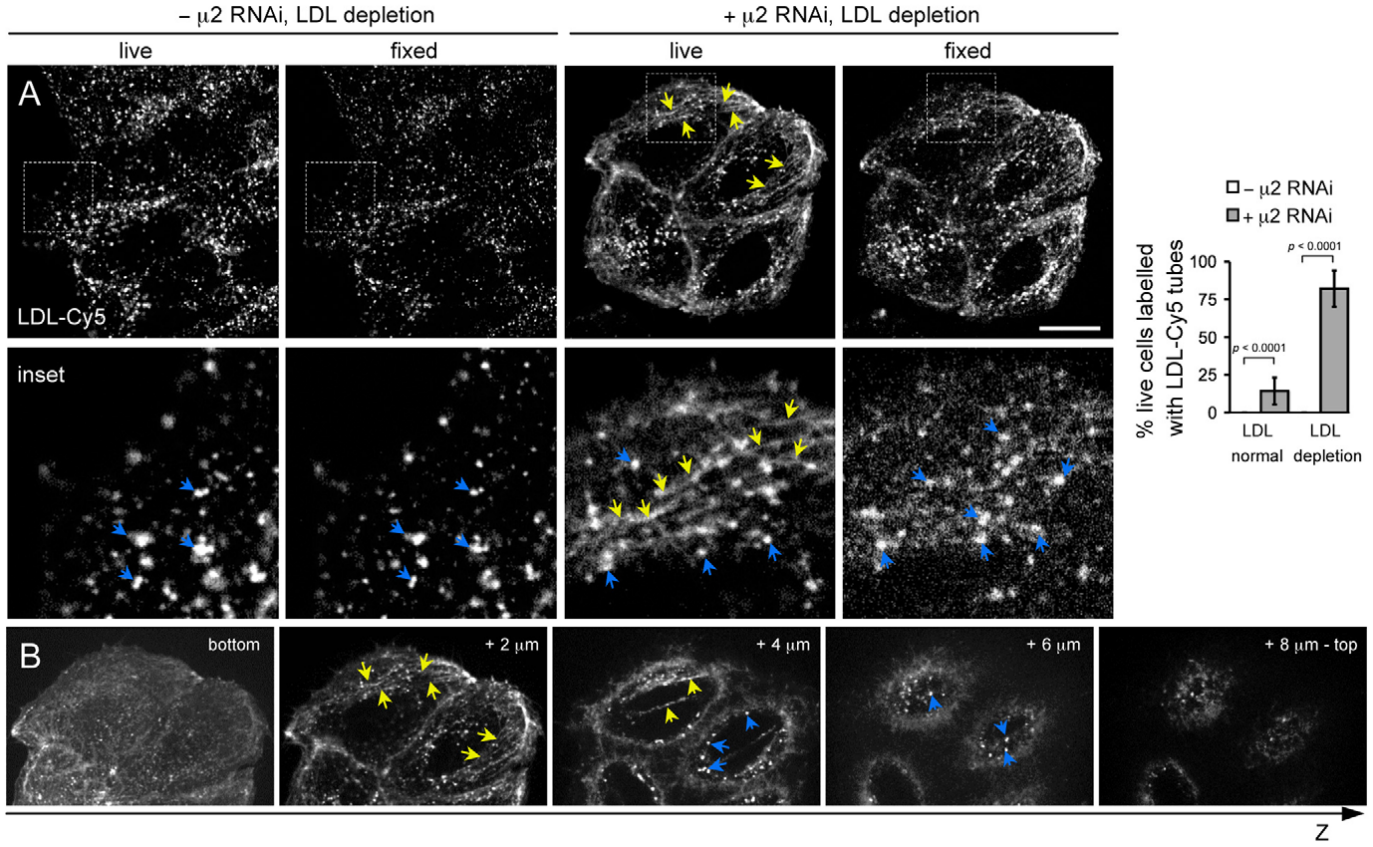
Enrichment of endocytic tubules labeled with LDL-Cy5 in HeLa cells depleted of AP-2 and LDL and their susceptibility to chemical fixation. (**A**) HeLa cells depleted (+ μ2 RNAi) or not (-μ2 RNAi) of AP-2 by treatment with μ2 RNAi were plated and incubated for 24 hrs in medium containing LDL-deficient serum (LDL depletion). The samples were then incubated with 10 µg/ml Tf-A488 (to identify AP-2 depleted cells – *not shown*) and 0.72 µg/ml LDL-Cy5 for 5 min, followed by live-cell imaging with the spinning-disk confocal microscope (live). The cells were then fixed directly on the microscope stage using 3.7% PFA for 10 min and imaged again using the same exposure (fixed). Yellow arrows highlight the presence of tubular structures containing LDL-Cy5 in AP-2 depleted HeLa cells. Blue arrows highlight fluorescent spots due to the endosomal accumulation of LDL-Cy5. The tubular structures are labile, as documented by their almost complete disappearance following chemical fixation. Bar, 20 µm (10 µm in the insets). Images are representative of 100 random cells acquired for each category from 3 independent experiments. The histograms show the fraction of live cells containing tubes labeled with LDL-Cy5 observed in 100 Tf-negative cells selected randomly from the imaging fields. Data was obtained from 3 experiments performed independently. Similar results were obtained using BSC1 cells (*not shown*). (**B**) Images from a Z-stack acquired with 0.3 µm steps showed the intracellular localization of the LDL-Cy5-labelled tubes (yellow arrows) and the presence of spots (blue arrows) within the cells displayed on ‘+ μ2 RNAi, LDL depletion, live’ on panel A.

### Western blot analysis

Total cell extracts were prepared as follow: BSC1 or HeLa cells grown on 10 cm^2^ dishes were lysed by addition of 150 µl of SDS-PAGE buffer, sonicated briefly (5 times 10 s), denaturated (10 min boiling) and spun (10 min, 14000 *g*). Total cell extracts were subjected to SDS-PAGE in MOPS buffer using 10% NuPAGE gels (Invitrogen, Carlsbad, CA) followed by transfer onto PVDF membranes. The membranes were probed with monoclonal antibodies specific for μ2- or α-adaptin and actin. The primary antibodies were detected by chemiluminescence using a goat anti-mouse coupled to HRP (Bio-Rad, Hercules CA). The signals on the blot were analyzed using Image J (NIH).

### Tf, LDL, CD8-LDLR and FM 1-43 Uptake assays

#### Uptake of Tf and LDL in full serum

Uptake of Tf-A594 or Tf-A647 (10 µg/ml) and LDL-Cy5 (0.72 µg/ml) (Figure 1 and Figure 5B, C) or 20 µg/ml of LDL-Cy5 (not shown) was followed in BSC1 or HeLa cells treated or not with siRNA specific for μ2-adaptin by using an imaging-based assay [13], [14]. Cells were plated on glass coverslips and grown overnight in complete medium (10% FBS). The uptake experiments were initiated by incubating the cells for 10 min at 37°C with the fluorescently labeled ligands diluted in imaging medium (α-MEM without phenol red supplemented with 20 mM HEPES, pH 7.4 and 5% FBS). Cells were then washed three times with ice-cold PBS (to remove unbound ligands), fixed with 3.7% PFA for 20 min at room temperature, and mounted with a mounting solution containing 0.01% p-phenylenediamine (1,4-Benzenediamine hydrochloride) and 10% polyvinyl alcohol (Mowiol). Image acquisition and analysis were done as described below.

#### Uptake of CD8-LDLR chimera

Uptake of the CD8-LDLR chimera (Figure 5A) was monitored by using as ligand the antibody specific for the ectodomain of CD8 used as in [15], except that the cells were always kept at 37°C both before and during the incubation period with the antibody. As a control, we also reproduced the apparent lack of AP-2 dependence of CD8-LDLR uptake observed by Motley and colleagues in experiments where the antibody binding was performed at 4°C instead of 37°C, After fixation the cells were permeabilized with 0.05% saponin, incubated with an Alexa Fluor 594-labelled goat anti mouse antibody in the presence of 1% FCS and mounted with the mounting solution described above. Image acquisition and analysis were done as described below.

#### Uptake of Tf and LDL after a 4°C pre-incubation step

In some of the uptake experiments, the cells were first incubated at 4°C during the adsorption of Tf-A594 and LDL-Cy5 and then warmed to 37°C (Figure 5D) as described in [15]. A 6-well plate containing Hela cells on glass coverslips was placed over wet ice, and the cells then washed with ice-cold serum-free medium (α-MEM without phenol red supplemented with 20 mM Hepes pH 7.4 and 0.1% BSA). The 6-well plate containing the cells was then placed on a rocker for 1 h at 4°C in serum-free medium supplemented with 10 µg/mL Tf-A594 and 0.72 µg/mL LDL-Cy5. Endocytosis was activated for 10 min at 37°C by replacement with pre-warmed complete medium (DMEM containing 10% FCS). Cells were then washed three times with ice-cold PBS, fixed with 3.7% PFA for 20 min at room temperature and mounted with the mounting solution described above. Image acquisition and analysis were done as described below.

#### Uptake of Tf and LDL in LDL-depleted cells

The effect of LDL starvation on cells prior to the uptake assay (Figure 5E) was followed in HeLa cells plated on glass coverslips as described in [6]. HeLa cells were grown on coverslips in DMEM supplemented with either 10% lipoprotein-deficient serum (LPDS, Cocalico Biologicals, Reamstown, PA) or 10% LDL-free serum (a gift of Prof. Monty Krieger, Massachusetts Institute of Technology, US) and 2 mM L-glutamine for 24 h, a procedure that up-regulates the number of LDL receptors. The cells were then incubated for 10 min at 37°C in the same LDL-deficient medium supplemented with 10 µg/ml Tf-A594 and 0.72 µg/ml LDL-Cy5. Cells were then washed three times with ice-cold PBS, fixed with 3.7% PFA for 20 min at room temperature and mounted with the mounting solution described above. Image acquisition and analysis were done as described below.

The effect of serum starvation on cells prior to the uptake assay (Figure 5F) was studied in HeLa cells plated on glass coverslips for 24 h in DMEM supplemented with 10%FBS. The cells were then incubated for another 6 h in DMEM supplemented with 0.1% FBS and 2 mM L-glutamine, followed by incubation for 10 min at 37°C with the same medium containing 10 µg/ml Tf-A594 and 0.72 µg/ml LDL-Cy5. Cells were then washed three times with ice-cold PBS, fixed with 3.7% PFA for 20 min at room temperature and mounted with the mounting solution described above. Image acquisition and analysis were done as described below.

#### Uptake of FM 1-43 dye

The effect of AP-2 depletion on the uptake of FM 1-43 styryl dye (Figure 6A) was studied in HeLa cells plated on glass coverslips for 24 h in 10% FBS. The cells were then kept for another 6 h in the same medium or in DMEM supplemented with 0.1% FBS and 2 mM L-glutamine, followed by incubation with 5 µg/ml FM 1-43 diluted in imaging medium for 2 min at 37°C. The cells were then quickly washed with pre-warmed imaging medium (to remove unbound FM 1-43) and imaged using the spinning-disk CSU-X1 confocal setup (see below). Alternatively, LDL-Cy5 (0.72 µg/ml) was used for a 5 min incubation period (Figure 6B). In this case, the cells were imaged live or immediately after fixation (3.7% PFA for 10 min) on the microscope stage.

### Fluorescence microscopy

#### Spinning-disk confocal live-cell imaging

Cells were plated on 25 mm diameter, #1.5 glass coverslips and grown in DMEM supplemented with 10% FBS. Before imaging, the medium was changed to α-MEM without phenol red supplemented with 20 mM HEPES, pH 7.4 and 5% FBS and imaging done with 5% CO_2_ and 100% humidity in a temperature-controlled sample holder (20/20 Technology, Inc.; Wilmington, NC) located inside an environmental chamber set at 37°C also containing the objective lenses.

Live-cell imaging data were acquired with a Marianas^TM^ system (Intelligent Imaging Innovations, Inc., Denver, CO) consisting of a spinning disk confocal head (CSU 22 Yokogawa, Japan) attached to a computer controlled spherical aberration correction device (SAC, Intelligent Imaging Innovations, Inc, Denvers, CO) and to a fully motorized inverted microscope (Axiovert 200 M, Carl Zeiss, Inc., Thornwood, NY) using a 63× lens (Pan Apochromat, 1.4 NA, Carl Zeiss, Inc) under control of SlideBook 4.2 (Intelligent Imaging Innovations Inc, Denver, CO). 16-bit digital images were obtained using a back-illuminated cooled CCD camera (Cascade 512B; Roper Scientific, Photometrics, Tuscon, AZ) with no binning. Three 50 mW solid-state laser (473 nm, 561 nm and 641 nm; Crystal Laser, Reno, NV) coupled to an acoustic-optical tunable filter (AOTF) were used as light source to excite EGFP, Tomato, Alexa594, Alexa647 and Cy5. Photo-bleaching was reduced by turning off the illumination during the readout period from the camera to the computer. Images were acquired at the free (top) or attached (bottom) surface of the cells with exposure times between 50 and 200 milliseconds according to established protocols [3], [4].

#### Epifluorescence imaging of fixed samples (uptake assays)

Wide-field fluorescence imaging of fixed cells was carried out using an Everest^TM^ system (Intelligent Imaging Inovations, Inc., Denver, CO) consisting of an upright microscope (Carl Zeiss, Inc) equipped with a manual spherical aberration correction device (SAC, Intelligent Imaging Innovations, Inc). 12-bit digital images were obtained with a cooled CCD camera (Cool Snap HQ, Photometrics, Trenton, NJ) with 2×2 binning under the control of Slidebook 4.1 (Intelligent Imaging Innovations, Inc, Denver, CO). 3D stacks of optical sections spaced 0.3 or 0.5 µm and spanning the complete volume of the cells were acquired using 63× or 40× oil immersion NA 1.4 lenses (Pan Apochromat, Carl Zeiss, Inc), respectively.

In some cases, images of live and fixed samples were acquired using a Nikon Eclipse TE-2000 (Nikon, Japan) equipped with a CSU-X1 spinning disk confocal head (UltraVIEW VoX, Perkin-Elmer, England) under control of Volocity 5.0 (Improvision, England). 16-bit digital images were obtained with a cooled EMCCD camera (9100–50, Hamamatsu, Japan). 3D stacks of optical sections spaced 0.5 µm were acquired using a 60× or 40× oil immersion NA 1.4 lens (Pan Apochromat, Nikon, Japan).

### Data analysis

#### Live-cell imaging data

Automated unbiased identification of fluorescent spots corresponding to all clathrin and AP-2 coated structures and quantitative tracking of their dynamics as a function of time using previously described criteria and procedures [3], [4]. Determination of descriptors such as lifetime and maximum fluorescence intensity was obtained using a Matlab 7 (Mathworks, Natick, MA) image analysis application [4], [16]. The panels in Figure 4 show log-log plots of the maximum fluorescence from AP-2 determined for individual spots immediately before uncoating as a function of the coated-structure lifetime. Analysis was carried out on objects for which the fluorescent signal appears and disappears within the span of the time-lapse series [3], [4], [16].

#### Fixed samples (uptake assays) data

The integrated amount of fluorescently tagged ligand accumulated within the cell boundaries corrected by background represents the total uptake of ligand. Briefly, for each cell a three-dimensional stack of images was acquired 0.3 µm apart using the spinning disk confocal microscope. The amount of internalized ligand is the integrated intracellular fluorescent signals (e.g. bright spots corresponding to endosomes) enclosed within the cell boundaries corrected by the background fluorescence signal around the cell as described before (Boucrot and Kirchhausen, 2007). Briefly, the integrated intracellular signals were determined from a 2D projection image of the fluorescence intensities of a Z-stack lacking the most bottom and top planes as they mostly contain plasma membrane signals. The cell outline in each plane was determined by increasing the brightness of the image. The integrated fluorescence within the cell was background-corrected by substraction of the fluorescence signal surrounding the cell under analysis and its value was then normalized to the mean (average) of the control cells set to 100.

Data is presented as mean ± standard deviation (SD) of at least 50 cells in each category, from 3 independent experiments. Statistical significances (*p* values) were calculated by unpaired two-tailed Student's *t*-test. *p* values inferior to 0.01 were considered statistically significant.

## Results

### AP-2 depletion by RNAi and inhibition of Tf uptake

The most effective reported procedure for depleting AP-2 complexes is a sequential protocol with a 48 h interval between transfections [5]. We used this protocol and confirmed by western blot analysis that residual μ2-adaptin had decreased to 2.5±1.8% and to 2.1±2.0% (*p*<0.0001, n = 3) in HeLa and BSC1 cells, respectively (Figure 1). Moreover, depletion of μ2-adaptin results in the instability of the AP-2 tetrameric complex and degradation of the α-subunit [5]. Indeed, α-adaptin is depleted to 1.5±1.4 and 0.4±0.1% of the levels in control Hela and BSC1 cells (Figure. 1). To confirm functional depletion of AP-2, we followed the effect of μ2 knockdown on the receptor-mediated endocytosis of transferrin (Tf), for which clathrin-mediated endocytosis is the predominant route of uptake [5]. Stable expression of σ2 adaptin fused to EGFP gives efficient incorporation into active AP-2 complexes, which produce a characteristic punctate pattern of fluorescence at the plasma membrane of the expressing cells [3], [4], [12], [16]. We incubated HeLa or BSC1 stably expressing σ2-EGFP cells with 10 µg/ml of Tf labeled with Alexa-594 (Tf-A594) for 10 min at 37°C, followed by a quick rinse at 4°C - a temperature that blocks endocytosis - to remove unbound Tf. The cells were fixed and imaged in 3D by wide-field fluorescence microscopy (Figures 2A and B). In control cells (-μ2 RNAi), σ2-EGFP is punctuated at the plasma membrane and Tf accumulates in characteristic endosomal punctae. In AP-2 depleted cells (+μ2 RNAi), σ2-EGFP becomes largely cytosolic and Tf remains at the cells surface and fails to internalize (Figures 2A and B). Thus, as expected and in full agreement with earlier observations [5]–[9], AP-2 is required for efficient Tf internalization by clathrin-mediated endocytosis.

### Every endocytic clathrin-coated structure contains AP-2

To determine the composition of residual AP-2 in coated pits under conditions of AP-2 depletion in BSC1 and HeLa cells, we used high sensitivity, live-cell, spinning-disk confocal imaging. AP-2 and clathrin were labeled respectively by stable expression of σ2-EGFP and by transient expression of the clathrin light chain, LCa, fused at its N-terminus to Tomato (Tomato-LCa) [3], [4], [12], [16], [17]. Using σ2-EGFP as a marker for live-cell imaging, we found, consistent with previous results [5], [9] and the assays in Figures 1 and 2, a large decrease in the number and intensity of the fluorescent AP-2 spots associated with the plasma membrane in virtually all RNAi-treated BSC1 and Hela cells (Figure 3 and 4). The images from control BSC1 cell (-μ2 RNAi in Figure 3A) illustrate both the accumulation of fluorescent Tf-A647 into punctate endosomal structures (left panel) and the complete colocalization of all AP-2 with clathrin fluorescent spots at the adherent (bottom) surface of the same cells (central panel) [3], [4], [12], [16], [17]. The kymograph (right panel) illustrates the dynamic behavior of single spots; each line corresponds to the gradual recruitment of AP-2 and clathrin within a single coated pit until a maximum fluorescence is achieved at the time of coated vesicle budding, followed almost immediately by uncoating and loss of signal. A similar experiment conducted with AP-2 depleted BSC1 cells after μ2 RNAi treatment (+μ2 RNAi in Figure 3A) shows the expected increase in the Tf signal at the cell surface (due to accumulation of the Tf receptor) and a substantial inhibition of transferrin uptake (Figure 3B). A single frame taken from the time lapse series recorded at the bottom surface of the same cell (central panel) and the corresponding kymograph (right panel) show a substantial reduction in the number of AP-2 and clathrin spots. This reduction is in agreement with the ∼10–20 fold decrease in the rate of coated pit formation as determined by the rate of nucleation (Figure 3C). It is important to note that all AP-2 spots colocalized with clathrin and all clathrin spots colocalized with AP-2 (∼300 spots from 5 cells in 3 independent experiments); we were unable to find clathrin spots at the plasma membrane devoid of AP-2.

For a quantitative assessment of the clathrin and AP-2 content of single endocytic coated structures and of the dynamics of these coats, we used data from time-lapse movies acquired from both attached (bottom) and free (top) cell surfaces. We first focused on canonical clathrin-coated pits, the rapidly forming structures that deform the underlying membrane by progressive recruitment of clathrin, adaptors and other regulatory proteins and ultimately bud inward to form coated vesicles [3], [4], [16], [18]. BSC1 cells plated for a few hrs on glass coverslips under standard tissue-culture conditions form only canonical endocytic coated pits, both on their top and bottom surfaces, with lifetimes typically between 30 and 90 s (Figure 4A and [3], [4], [18]). In these structures, the maximum fluorescence is proportional to the number of clathrin or AP-2 molecules contained within the completely assembled coat (Figure 4A and B); in the case of clathrin, it also reflects the relative size of the final coat [3], [4], [16], [18]. Fluorescence from clathrin and from AP-2 in endocytic coated pits of cells expressing similar amounts of clathrin tagged with Tomato-LCa was significantly weaker (∼2-fold and 4-fold, respectively, both *p*<0.0001) in siRNA treated cells than in control cells (Figure 3E and 4B), suggesting that coated pits are on average smaller when AP-2 is severely limiting. As emphasized above, we never found a clathrin spot that completely lacked AP-2. Similar results were obtained when the AP-2 depletion experiments were carried out with HeLa cells stably expressing σ2-EGFP and transiently expressing Tomato-LCa. As in BSC1 cells, μ2 knockdown dramatically reduced the number of endocytic coated pits (Figure 4C and D; *p*<0.0001, 3 cells). In each case, every detected spot contained less clathrin and AP-2 than the coated pits structures in control cells (Figure 4F; *p*<0.0001, n = 50 pits). Again, there were no instances of endocytic clathrin-containing pits devoid of AP-2. While canonical clathrin-coated pits and vesicles form at the free (top) and adherent (bottom) surfaces of HeLa cells (Figure 4C and D), larger, less dynamic, longer-lived structure, referred to as clathrin-coated plaques, also form at the bottom surface (Figure 4C) [4]. These structures contain less sharply curved coats; their clathrin lattices do not close off, but instead move uniformly inward from the cell surface shortly before membrane fission. In HeLa cells with depleted AP-2 we also observed coated plaques at the adherent surface; like the coated pits, they also contained reduced amounts of both AP-2 (Figure 4C) and clathrin (not shown) relative to controls, but some AP-2 was always present (Figure 4C). We conclude from the entire series of experiments that some AP-2 is required to sustain the formation of endocytic, clathrin-coated structures. This conclusion is consistent with the idea that AP-2 participates in the earliest steps of coat assembly [3], [16].

### AP-2 depletion inhibits LDL uptake

Clathrin mediated endocytosis is the only known pathway of LDL uptake, and clathrin depletion by RNAi blocks transferrin internalization ([5]–[9]; this study). The endocytic motif for LDLR sorting to coated pits is the FDNPVY sequence in its cytosolic tail [19]. This motif interacts directly with AP-2 [20], [21], and also with the PTB-like domains of Dab2 and ARH [22], [23], which in turn bind both AP-2 and clathrin. The insensitivity of LDL uptake to AP-2 knockdown [5] has led to the proposal that it depends on Dab2 and ARH, but not AP-2 [6], [7]. The evidence adduced for AP-2 independence was the observation that AP-2 depletion by RNAi does not prevent endocytosis of the chimeric protein CD8-LDLR composed of the CD8 ectodomain and its transmembrane region fused to the cytosolic tail of the LDL receptor [5].

In the experiments cited in the preceding paragraph, endocytosis was measured following a pretreatment designed to increase the amount of detectable LDL internalized by cells. In some cases [5], [6], [8], cells were cooled to 4°C during an adsorption step to increase binding of LDL to its receptor, then warmed to 37°C to activate endocytosis; in other cases [6], [7], cells were serum- or lipoprotein starved to increase the number of available LDL receptors on the cell surface [24]. Either treatment could have the effect of increasing the likelihood that LDL receptors will be captured by the residual coated pits remaining in AP-2 depleted cells, such as those detected in the experiments reported here. In fact, for EGFR, it is known that uptake is strongly decreased by AP-2 RNAi if cells are maintained at 37°C throughout the experiment but that introduction of a cooling step renders uptake insensitive to AP-2 knockdown [10]. We therefore carried out the following experiments. (1) We used the same CD8-LDLR fusion protein previously used by Motley and colleagues [5] and the same type of cells (HeLa) in which these experiments were performed. We confirmed their results by uptake of CD8-LDLR in AP-2 depleted cells monitored by pre-incubation at 4°C with the antibody specific for the ectodomain of CD8 and then warmed to 37°C for 10 min (not shown). (2) We then eliminated the cooling and warming steps and followed the uptake of CD8-LDLR in cells kept at 37°C. AP-2 knockdown reduced CD8-LDLR endocytosis and Tf uptake to similar extents (Figure 5A). (3) The receptor-mediated uptake of fluorescent human LDL labeled with Cy5 by BSC1 or HeLa cells depleted of AP-2 was also inhibited when the cells are kept at 37°C (Figure 5B and 5C); in this case most of the LDL signal remains at the cell surface. In contrast, as similar to the observations with CD8-LDLR, LDL is internalized in AP-2 depleted HeLa cells first incubated at 4°C with LDL-Cy5 and then warmed at 37°C (Figure 5D). (4) When we monitored the uptake of fluorescent LDL particles into AP-2 depleted HeLa cells that had been subjected to lipoprotein starvation by incubation with LDL-depleted serum or with medium supplemented with 0.1% serum, we found an almost normal LDL uptake (Figure 5E and 5F), even though transferrin uptake was fully inhibited. The increase in LDL binding at the cell surface of LDL-depleted cells with respect to control cells (8.1±2 times, p<0.0001; 50 cells) was consistent with massive upregulation of cell-surface LDL receptor. In the lipoprotein-starved cells, AP-2 depletion by μ2-RNAi also led greater than 10-fold decrease in the number of clathrin and AP-2 structures, and in all cases these clathrin coats contained at least some AP-2 (4 cells, n = 150 events). Although the overall clathrin-mediated uptake of transferrin and the number of active coated pits were similarly impaired by AP-2 knockdown, it is possible that following lipoprotein starvation, increased concentration of LDL receptors leads to more efficient use, for LDL uptake, of the residual clathrin- and AP-2 containing endocytic structures.

### LDL uptake in AP-2 depleted cells might follow a tubular endocytic route

An alternative explanation for the apparently normal level of LDL uptake in lipoprotein-starved AP-2 depleted cells is that its endocytosis follows a different route. This possibility was first tested by using live-cell imaging to follow the uptake of a general endocytic marker, the membrane impermeable dye FM 1-43, in a mixture of lipoprotein-starved control HeLa cells and cells depleted of AP-2 by 3-days treatment with the μ2 siRNA. FM-1-43 rapidly becomes fluorescent when bound to membranes [25], and labeling of intracellular endosomal structures is a direct reflection of its uptake [14], [26], [27]. As shown before, uptake of FM 1-43 in membrane-bound carriers led to the rapid appearance of a large number of intracellular punctae structures, most likely endosomes, within 5 min of addition to the medium (Figure 6A, top insets, - μ2 RNAi). In contrast, AP-2 depleted cells imaged in the same sample had long fluorescently labeled tubes emanating from the plasma membrane indicative of the continuity between the plasma membrane and the tubes (Figure 6A and B, bottom cell; see also the images from selected planes in Z-stack and + μ2 RNAi insets and Movie S1) in addition to the conspicuous endosomal spots (Figure 6A and B, blue arrows). Such tubes were less abundant in AP-2 depleted cells kept in normal serum (Figure 6A, LDL normal and histogram). We tested whether similar tubes might contain LDL-Cy5 in AP-2 depleted and lipoprotein-starved cells. Indeed, after a 5 min incubation with LDL-Cy5 at 37°C, most lipoprotein-starved and AP-2 depleted HeLa cells showed staining of tubes that seemed to reach the cell surface (Figure 7A and B, + μ2 RNAi, live; see also selected image planes in the Z-stack and summary data provided in the histogram) in addition to the expected punctae endosomal pattern (Figure 6A, blue arrows). In contrast, almost no cells displayed such tubes if they contained normal amounts of AP-2 (Figure 6A, - μ2 RNAi, live and histogram). We believe that these tubes were missed in earlier observations of chemically fixed samples, because their integrity seems to be extremely susceptible to chemical fixation, as highlighted by their apparent absence in the LDL uptake experiments shown in Figures 5E and 5F. To explore this possibility, we compared the distribution of LDL-Cy5 in the same AP-2 depleted and lipoprotein starved HeLa cells before and after fixation with 3.7% PFA. The images in Figure 7A show that most of the tubes labeled with LDL-Cy5 had disappeared within 2–3 min of chemical fixation, leaving instead only the endosomal punctate staining (Figure 7A, ‘+μ2 RNAi, LDL depletion, fixed’). These results suggest that entry of LDL receptors into lipoprotein-starved and AP-2 depleted cells occurs through a previously unrecognized tubulo-vesicular endocytic pathway that becomes more prevalent in cells depleted of AP-2.

## Discussion

### All endocytic clathrin coats contain AP-2

Our central observation is that despite a very large decrease in cell-surface AP-2 following RNAi knockdown of μ2 adaptin, there is always a detectable amount of AP-2 in the clathrin coated pits that continue to form (at greatly reduced frequency) in the RNAi-treated cells. These clathrin coats are smaller (lower final clathrin intensity) than the average of those in untreated cells. Our results indicate that AP-2 is an essential component of an endocytic clathrin coat. They also suggest that AP-2 participates in initiation of coat assembly. The only well documented examples of plasma-membrane clathrin structures lacking AP-2 are the extended clathrin patches associated with invading *Listeria monocytogenes* and with phagocytic uptake of polystyrene beads coated in InlB, the ligand required for invasion [28]. These clathrin patches are much larger than conventional clathrin-coated pits and have quite different dynamics.

AP-2 depletion, as previously reported, leads to large decrease in the number of endocytic clathrin coated structures. Under extensive RNAi treatment of HeLa cells, in which knockdown is most effective, we observed more than 20 fold reduction in the number of clathrin coated structures. These structures were dynamic, and they all contained AP-2. This was not appreciated in earlier experiments, as they were all based on imaging of fixed samples. More important, we could not detect a single example of a clathrin coat at the cell surface that lacked AP-2. So even under conditions of severe AP-2 depletion, when the amounts of clathrin and (presumably) other adaptors remain normal, endocytic clathrin coats always contain some AP-2. These data suggest an important role of AP-2 during formation of clathrin coats.

### Inhibition of LDL uptake in AP-2 depleted cells

It is well established that clathrin coated structures are the principal endocytic carriers for the first step of internalization of transferrin or LDL. Thus, it is not surprising that AP-2 depletion results in the severe inhibition of Tf uptake, documented by others and confirmed here. An apparent contradiction to the AP-2 requirement for all clathrin-mediated uptake has been the apparently normal uptake of the CD8-LDLR chimera in cells depleted of AP-2, despite the reduced uptake of this ligand in cells depleted of clathrin (Motley *et al.*, 2003). A logical conclusion has been that other adaptors, such as Dab2 and ARH, sort the LDL receptor to the few clathrin coats that still form in AP-2 depleted cells [6], [7].

We now find that CD8-LDR or LDL uptake is blocked in cells depleted of AP-2 if internalization is monitored in cells maintained throughout the experiment at 37°C and in a medium with 10% serum. The decrease in uptake is consistent with the ∼10–20 fold decrease in the number of available coats forming under these conditions. This assay differs from the previous one in that it corresponds to a steady-state uptake measurement rather than a single round of endocytosis (immediately after the warm-up step). The normal levels of CD8-LDLR or LDL uptake observed if ligand binding is done at 4°C (a commonly used method for enhancing association with the LDL receptor), could be explained if CD8-LDLR or LDL receptors diffusing on the membrane are efficiently captured by partially formed coated pits, assembly of which is very slow at the low temperature. (Note that the warm-up experiment is essentially a pulse chase, showing events that have already occurred when endocytosis is triggered at the higher temperature. Because of thermal instabilities, we have not been able to follow the process from low to high temperature by live-cell imaging, to confirm the suggested kinetics.) The uptake of EGF is likewise sensitive to AP-2 depletion if the internalization assay is done at 37°C, whereas almost no inhibition is observed when the internalization is preceded by a 4°C binding step [10].

### An alternative entry route for LDL in lipoprotein starved and AP-2 depleted cells

A common protocol used to follow LDL uptake involves use of cells first exposed to medium containing low amounts of LDL by either serum starvation or LDL depletion [6], [7]. These procedures, which induce accumulation of excess LDL receptor, might lead to more efficient use of the residual clathrin-mediated traffic, thereby accounting for the apparent insensitivity of LDL uptake to AP-2 depletion by μ2 RNAi treatment. It is also possible that the accumulated receptor can follow a clathrin- and AP-2 independent route when the frequency of coated-pit formation is low. We show here that LDL is contained in tubular invaginations connected, directly or indirectly, with the cell surface. Furthermore, our data indicates that the invaginations are very sensitive to chemical fixation, a probable explanation for why their abundant presence was not noticed before in LDL depleted cells. The invaginations are not induced by re-exposure of the cells to LDL, and they are readily accessible to the surface, as indicated by their rapid labeling with the FM 1-43. Similar tubular invaginations, involved in the traffic of cholera toxin [29], the major histocompatibility class I (MHC-I) molecules [30] and shiga toxin [31] and with access to endosomal compartments have been noted before using live cell imaging, but little is known about the molecular basis for their formation. It remains to be determined whether Dab2, the LDLR-specific cargo adaptor [6], [7] mediates the presence of LDLR in the tubules.

We have observed that clathrin depletion by RNAi treatment of cells starved of LDL results in a substantial decrease in the surface staining of LDL receptors as determined by immunofluorescence and also in a significant reduction in the number of deeply invaginated tubules containing LDL (not shown). The combined reduction in the number of LDL receptors located at the cell surface and in the number of tubules is therefore fully consistent with earlier observations showing that clathrin depletion significantly decreased the efficiency of LDL internalization during conditions of LDL-starvation [6], [7].

### Conclusion

In summary, our data show that AP-2 complexes are necessary for the formation of endocytic clathrin coats. Our results also eliminate any need to invoke a bypass of AP-2 by alternative adaptors that might function through the endocytic clathrin coated pit/coated vesicle route to explain LDL entry in cells depleted of AP-2. Finally, we show that under certain conditions of stress, cells can upregulate alternative endocytic structures with the potential to provide compensatory trafficking pathways.

## Supporting Information

Movie S1 3D image stacks of HeLa cells depleted of AP-2 and briefly incubated with FM 1-43 dye. The movie contains sequential optical sections spaced 0.3 microns apart from the series shown in Fig. 6B. The movie goes through sections from the top to the bottom of the cells. Tubular structures projecting into the cell interior are clearly observed in the sections closest to the bottom of the cells depleted of AP-2 by μ2 RNAi treatment. These tubules are hard to observe in cells containing normal amounts of AP-2.
(5.84 MB MOV)
